# The 5Rs of Rugby: A qualitative evaluation of the development, delivery, and experience of a mental health literacy and social norms intervention with elite rugby union players in Ireland

**DOI:** 10.1371/journal.pmen.0000258

**Published:** 2025-09-24

**Authors:** Robert C. Dempsey, Deirdre Lyons, Philip Clarke

**Affiliations:** 1 School of Psychology, Faculty of Health and Education, Manchester Metropolitan University, Manchester, United Kingdom; 2 Rugby Players Ireland, Dublin, Ireland; 3 College of Health, Psychology and Social Care, University of Derby, Derby, United Kingdom; Hamdard University - Islamabad Campus, PAKISTAN

## Abstract

Elite rugby players are as likely to experience mental health challenges as the general population yet face unique pressures on their wellbeing associated with competitive sport. Few mental health literacy interventions have been conducted with elite rugby professionals, particularly those which consider the nature of rugby as a demanding, competitive team sport with unique group dynamics within teams. We conducted a novel social norms and mental health literacy project with current elite players contracted to the provincial rugby union teams in Ireland. The project, developed in collaboration with Rugby Players Ireland, featured a mental health literacy workshop intervention and co-produced poster materials detailing the ‘5Rs of Rugby’, relating to player mental health, sources of support, and social norms feedback from an earlier survey, with messages focusing on players’ intentions to signposting teammates to formal and informal sources of support. We report a qualitative evaluation of the development, delivery, and the experience of participating in the intervention based on focus groups, a survey, and interviews with staff and players whilst considering the context of elite rugby. An inductive reflexive thematic analysis identified three themes relating to (i) *Rugby: ‘fickle and all-encompassing’*, (ii) *Trust and Brotherhood*, and (iii) *Experiences of the 5Rs of Rugby intervention.* Participants felt that the intervention engaged the players, provided a psychologically safe environment for mental health discussions and disclosures, and reinforced existing positive social norms around help-seeking. Our analysis identified several challenges and considerations for supporting players’ mental health particularly around longer-term awareness campaigns, focusing on prevention, and providing actionable support for players.

## Author summary

Professional rugby union players experience similar mental health challenges like the rest of the population but also have to manage the demands and the competitive nature of professional rugby which can put unique pressures on their mental health. Our past research and experience working with professional players suggested some mixed levels of mental health literacy amongst Rugby Union professionals and some low levels of help-seeking for mental health challenges. We conducted an intervention project to promote help-seeking behaviours amongst professional rugby union players in Ireland focusing on challenging and promoting help-seeking as a social norm (i.e., something most players would do). The current study explores player and staff experiences of taking part in this intervention based on an analysis of interviews, discussions, and an online survey, with players and staff involved in the project. Our social norms intervention was positively experienced by staff and players who expressed some surprise at the high level of support players would provide for their teammates who might be experiencing mental health challenges. Players viewed mental health focused interventions as needing to be more than one-off workshops and needing to provide a safe space for players to discuss and disclose their mental health experiences (e.g., away from team coaches).

## Introduction

Elevated levels of psychological distress and low wellbeing have been identified in elite rugby players compared to the general population [1,2]. Comparatively few studies have focused on current (active) elite rugby union players’ mental health outcomes, with most studies tending to sample retired players, where high rates of common mental health conditions have been reported particularly for those who were dissatisfied with their rugby career [3,4]. In terms of current players, the recent experience of anxiety and depression (30%), eating disorders (23%), overall psychological distress (18%), adverse alcohol use (15%) and sleep disturbance (13%) has been reported in a survey study of 990 active professional players across rugby codes (Union, League, 7s) [5]. A two-year study of elite male rugby league players in the British Super League reported higher rates of common mental health difficulties (anxiety and depression) in comparison to same-age men in the UK general population [6]. Current elite rugby union players also report high levels of psychological stress [7,8], both sport- and non-sport-related (e.g., interpersonal relationship stress), which can fluctuate over the course of a season and be more acute on training days compared to rest and match days [6,9,10]. Elite rugby can clearly pose unique stressors and demands on athletes’ mental health and their overall wellbeing, and so interventions which promote rugby athletes’ mental health, whilst accounting for the nature of rugby as a professional sport, are needed.

There is also evidence of low mental health literacy, high perceived stigma, and low mental health help-seeking intentions amongst elite rugby players [6,11,12]. Players are particularly reluctant to seek help from club-affiliated staff compared to other sources of support like friends, family, other teammates, and mental health professionals [13]. Research also suggests that increasing rugby union players’ mental health knowledge/literacy alone may not be sufficient to promote help-seeking, rather other psychosocial factors may be important to consider, particularly the influence of help-seeking related social norms [14]. There is an ongoing need for mental health interventions which promote elite athletes’ understanding of mental health related experiences, which normalise and reduce the perceived stigma of such experiences, whilst promoting help-seeking behaviours and accounting for unique group dynamics in elite sports teams, particularly rugby union [11]. To address this gap in knowledge, we conducted the first co-produced social norms intervention [15] for mental health help-seeking in a professional sport setting and the first such intervention for professional rugby union players. We report a novel qualitative evaluation of staff and players’ experiences of designing and delivering this intervention. It should be noted that rugby union is the main professional code played in Ireland, and all subsequent references to rugby in this article are in relation to rugby union, unless stated otherwise.

### The 5Rs intervention

As informed by our initial qualitative research [11], we conducted a collaborative project focusing on elite rugby players’ mental wellbeing and help-seeking behaviours. This was a season-long project featuring multiple survey waves assessing player wellbeing, functioning, team environment (e.g., psychological safety), as well as measuring players’ help-seeking related behaviours, norms, and intentions [16]. Key to the project was the delivery of mental health workshops to each of the professional provincial rugby men’s teams in Ireland as part of the intervention. The workshop approach was directly based on an existing intervention framework used to develop mental health literacy workshops for staff working in high-performance sport settings in Australia [17].

Each provincial club took part in one ‘5Rs’ workshop which focused on players’ mental health literacy and help-seeking behaviours. The workshops followed a structure based on the ‘5Rs of Rugby’, including: *Recognise*, a presentation and group discussion on recognising the signs of mental health challenges; *Reach Out*, small group player-led discussions on initiating mental health conversations and support with teammates; *Refer*, a presentation led by one of the clinical service staff on pathways to support available to players (e.g., Rugby Players Ireland’s (RPI) wellbeing service, team doctors); *Remain Supportive*, player-led small group discussions on players’ preferred mental health support strategies within their clubs and amongst team-mates; and the *Realities of Rugby*, information on support available via clinical psychology staff plus the development of the 5Rs poster by players including presentation of the social norms feedback to the group. Each provincial club developed their own 5Rs poster, which was later printed and displayed in prominent locations in their club facilities following the workshops. For further details of the development of the intervention approach and the workshop format, please see our reflective case study paper [16].

The workshops were led by staff from Rugby Players Ireland’s clinical service and supported by the respective club’s Player Development Manager (PDM), with attendance limited to that club’s players (i.e., no club staff were present at the workshops). The PDM role aims to promote the personal development and wellbeing of players by empowering players to take ownership of their own development both on and off the sporting field [18]. The PDMs involved in the current project work for Rugby Players Ireland and are based at each of the provincial clubs in Ireland, supporting a range of initiatives relating to player wellbeing and personal development, and are well-known to players and staff at the clubs. We limited attendance in the workshops to players and RPI staff given the mental health focus and aims to promote conversations amongst players about wellbeing and social norms within the team.

In line with the Social Norms Approach [15,19], the group social norms feedback was based on players’ responses on a prior baseline survey (in terms of players’ actual likelihood to support other teammates formally and informally, and the perceived rates of these behaviours amongst the team as part of the *‘Realities of Rugby’* section of the workshop). The Social Norms Approach aims to promote positive behaviours (health-related or otherwise) through the delivery of actual, credible, feedback highlighting what the majority of a social group do or think about a given behaviour or issue, thereby challenging any misperceptions of the actual norm for that behaviour [15,20]. By challenging misperceived norms or reinforcing existing positive norms of the majority [21], this reduces the often-subtle social pressure to conform to a (perceived) majority negative behaviour [15]. Social norms feedback can be delivered in different ways, from group-based approaches (e.g., social marketing, group discussions) to more individual personalised normative feedback (typically delivered via computer), as appropriate to the target group in consultation with key stakeholders and with the target group themselves [15,22–24]. For this project, the social norms survey items and feedback were based on existing social norms studies and interventions conducted for other health-related behaviours [25–30].

We adopted a group approach to the intervention and normative feedback presentation to encourage discussion of mental wellbeing and help-seeking amongst the players. This approach has worked effectively in our prior Social Norms Approach work in high schools, where a group-based activity was effective in challenging students’ misperceived norms of unhealthy snacking behaviours and encouraged the discussion of related peer norms [28,31]. The social norms data was collected in the present study’s baseline survey and informed the workshop feedback messages. The baseline data indicated that the significant majority of players would personally support their teammates informally (on a one-to-one basis) and would refer their teammate to a formal source of support (e.g., a medical professional, player wellbeing service), and that a significant majority of other players were perceived to do the same. That is, there was no clear gap between perceived and actual norms, with both informal and formal help-seeking being significant majority behaviours. Given the lack of a significant norms ‘misperception’, the norms feedback was used to facilitate a player discussion of mental health literacy, reinforce the existing strong positive social norms amongst the players, and discuss how players would support each other over the course of the rugby season.

The final part of the workshop activities involved each club co-producing a ‘5Rs of Rugby’ poster unique to their province which outlined the support for players’ mental wellbeing. These posters were tailored to each club’s identity, featuring their logos, photographs of players wearing their team colours, and were later displayed in prominent locations in each club (e.g., in training areas, changing facilities). Like the workshop structure, the ‘5Rs’ on the posters extended the ‘4Rs’ structure featured in Sebbens et al.’s (2016) *Mental Health in Sport* workshops to include a novel ‘*Reality in Rugby’* component focusing on players’ mental health experiences in the context of professional rugby [17]. The ‘5Rs’ poster included sections labelled: ‘*Recognise’* (statistics about the prevalence of mental health and wellbeing challenges); ‘*Reach Out*’, which was unique to each province (i.e., how to support teammates and manage one’s own mental wellbeing); ‘*Refer’* (who to speak to in relation to mental wellbeing concerns, including the contact details for formal support and the club’s PDM); player-developed sections on how to ‘*Remain Supportive*’; and finally ‘*The Reality in Rugby*’ which included content on how elite rugby might influence player wellbeing and how players have sought help, including the norms feedback (16).

### The present study

The aim of this study was to provide a qualitative exploration of player and staff experiences engaging in the 5Rs project. We are unaware of any existing studies which have developed and implemented a Social Norms Approach intervention with professional athletes, and so this project represents the first use of this approach in an elite sporting environment. There has also been a general lack of qualitative evaluations of social norms interventions in the broader research literature; such evaluations are important for understanding participants’ subjective experience of these interventions, identifying good practice and areas for development [15,31]. Therefore, the current study represents the first qualitative evaluation of this novel type of mental wellbeing and social norms-focused intervention conducted in an elite sporting environment with professional athletes.

In the present study we aimed to understand player and staff experiences of developing and participating in the intervention work described previously. We used a combination of qualitative methods, including focus groups, interviews, and surveys with players and staff who were involved in the project, to evaluate the delivery of the intervention considering the professional rugby environment and player and staff experiences. Based on this feedback, we also aimed to identify how such interventions with elite athletes can be developed and delivered in the future. We would highlight that the qualitative nature of this study does not accommodate conclusions about the effectiveness of the intervention to be drawn in terms of specific behaviour or attitudinal change. We, as a research team, have separately reflected on the processes and delivery of this intervention project elsewhere [16].

## Materials and methods

### Design and sample

Elite professional rugby players from four Irish provincial men’s teams (Connacht, Leinster, Munster, and Ulster) (n = 14) and staff from Rugby Players Ireland (n = 5, including Player Development Managers, PDMs, and clinical service staff who were involved in the project delivery) participated in the current study. Staff and players took part in separate focus groups (n = 3; 1 staff discussion, 2 player discussions), with a one-to-one interview conducted with a clinical psychologist involved in RPI’s player wellbeing service who delivered the mental health literacy component of the workshops (interviews and focus groups were conducted between late September 2023 to late November 2023). Three additional players from the men’s teams provided feedback on their experiences of the project via an anonymous online Qualtrics survey (these players did not participate in the focus group discussions). Small group discussions were deemed appropriate for the players and PDMs to facilitate interactions and sharing of experiences of the intervention activities and considering that these groups already existed. The one-to-one interview approach used with the clinical staff was appropriate given their different perspective and role at RPI in terms of their leading of some of the workshop content and given that this was the first occasion this specific member of staff had met some of the players face-to-face. Based on feedback from Rugby Players Ireland, and to accommodate other players who were unable to attend the group discussions, we used the anonymous online survey to capture any additional feedback (noting that the survey used the same question prompts as used in the group discussions). The combined approach across these different methods is consistent with the flexibility of reflexive thematic analysis [32], and so we pooled the data into one corpus for analysis.

All of the players involved in the broader intervention and this specific study were full professionals contracted to one of the men’s Irish provincial teams. Given the high-profile, potentially identifiable, nature of the player and staff participants in this study we cannot provide further details on the sample demographic characteristics to maintain the confidentiality of their data in line with our institutional research ethics approvals.

### Materials

Focus group discussions and interviews followed a semi-structured schedule, with an adapted version of these questions forming the basis of the anonymous online player survey (See Supplementary File). The discussions/interviews and the survey included the 5Rs of Rugby co-produced posters as a prompt for discussing the materials developed as part of the intervention (with players in the survey and discussion groups presented with their respective province’s 5Rs poster).

### Procedure

The focus group discussions and interviews were advertised by RPI and held online via Microsoft Teams using video recording and automated transcription with the discussions facilitated by the lead author. RPI arranged for private rooms for the players to join the discussion, with the staff discussions and interview held completely online given the geographical spread of the RPI staff across Ireland. Participants provided written consent to take part in the discussions after receiving participant information sheets, with verbal consent also confirmed at the start of the discussion recording. The recordings were stored on a secure institutional cloud storage to allow for the transcripts to be checked for accuracy and anonymised, edited by the first author to ensure they represented a verbatim account of the discussions (e.g., correcting errors with the automated transcriptions), with the recordings deleted upon production of the transcripts. The discussions and interviews lasted between 42–56 minutes. The player survey was hosted on Qualtrics and was anonymous in nature with participant consent taken via an explicit opt-in electronic form. The link to the survey was shared with players via their club’s PDM. Institutional research ethics approval for this study was obtained from Manchester Metropolitan University’s Faculty of Health and Education Research Ethics Committee (EthOS ID: 50179).

### Data analysis

Transcribed data from the focus groups, interview, and survey responses, were subjected to an inductive reflexive thematic analysis [33]. The transcriptions were analysed from a critical realist perspective accounting for the shared experience of the intervention project as mediated through participants’ perceptions of this experience [34]. The analysis consisted of six phases, including (i) data familiarisation, (ii) coding, (iii) the generation of initial themes, (iv) the development and reviewing of themes, (v) refining, defining, and naming themes, and (vi) the writing up of the themes in the present paper [35]. The lead author (RD) used the Microsoft Teams recordings to familiarise himself with the data alongside the written survey responses. Coding was primarily, but not exclusively, semantic in nature, with codes identified by RD and organised using nVivo version 14 [36]. Given the inductive nature of our analysis, we were unconcerned with practices like triangulation and member checking which would take a fully realist or positivist view of the data as opposed to our more critical realist approach. Such practices can be problematic for reflexive thematic analysis (for a discussion, we would recommend reading [37,38], especially considering RD’s active role in generating the codes and themes in the analysis (as discussed further in the *Reflexivity* section). To enhance the rigour of our analysis, there was an ongoing process of repeated reference back to the raw data throughout the analysis phases, with themes developed through several iterations of coding and re-coding of the data, and the re-reviewing of theme definitions. The initial clustering of codes and preliminary themes were discussed and reviewed by all authors through regular meetings prior to the finalisation of theme names and content.

### Reflexivity

RD is a Caucasian male researcher from England with an academic background in mental health, social psychology, and designing and conducting Social Norms Approach research and interventions [15,28,39]. RD had no prior experience working with athletes in an elite sporting environment and took leadership of this qualitative evaluation considering the other authors either worked for RPI (DL) or work as a registered Sport and Exercise Psychologist with athletes from various competitive sports (PC). Whilst RD had less of a working knowledge of professional rugby, he could take a more independent perspective, ensuring a data-led, inductive, analysis. In contrast to the other researchers who are both Irish and have detailed knowledge of rugby (DL, PC) and are either known to the players (DL) or familiar with their identities (PC), RD has a limited understanding of professional rugby (i.e., is an irregular viewer of televised games) and is unfamiliar with the identities of the specific players who participated in the broader project.

RD kept a reflexive journal over the course of the project summarising key impressions from each focus group or interview and tracking the data analysis progress. During the analysis, RD paid particular attention to his prior knowledge and experience with social norms studies and his (pre-analysis) expectations that players would have expressed interest in the novelty of the social norms aspect of the intervention whilst also expecting discussion of the challenges with player engagement and recruitment, and some mixed levels of mental health literacy amongst the players. Throughout the data (interview/discussions and survey), there was a notable focus on life as a professional rugby athlete and the interpersonal relationships within the teams and between players and RPI staff, particularly players’ need for security and a sense of safety when disclosing mental health challenges. RD noted players’ frank discussion of mental health workshops and the perceived limitations of workshops, which were often viewed sceptically as one-off activities delivered by outsiders to their teams. For all three researchers, the lack of a misperception gap between players’ actual and perceived norms for informally and formally supporting teammates was notable (i.e., players reported high levels of intentions to support teammates and perceived a similarly high majority would do the same) and surprising given that such protective behaviours tend to be underestimated [15].

## Results

Three key themes were identified through the reflexive thematic analysis which detail the social and sport-specific influences on player wellbeing and their experience of and engagement with the 5Rs intervention. First, *Rugby: ‘fickle and all-encompassing*’, describes life as a professional rugby player, the demands of rugby as a sport, the team culture and broader environment, and the influences on player wellbeing including on-field performances. This first theme captured the broader contextual influences on player mental health and wellbeing, not limited to on-field performance, highlighting key factors to consider when developing interventional work of this nature. The second theme, *Trust and Brotherhood*, captures the importance of team solidarity and togetherness, and the need to provide psychologically safe environments to support player wellbeing and permit players to be vulnerable when disclosing mental health challenges. The first and second themes demonstrate the importance of understanding the context of elite sport, the norms, cultures, and group dynamics of these teams, describing factors which influenced the later development and delivery of the 5Rs intervention and player engagement. The *Trust and Brotherhood* theme sat between the first and third themes (the latter focusing explicitly on the experiences of the intervention project we conducted). This theme highlighted the central importance of trust and the relationship between the players for their sporting performance, team functioning, players’ overall wellbeing, and their engagement in the 5Rs intervention, workshops, and poster materials developed in this project.

The third theme, *Experiences of the 5Rs of Rugby intervention*, describes player and staff experiences of the intervention itself, their evaluations of the project materials and aims, and discusses suggestions for the future development of such interventional work in the Irish rugby context. Several paradoxes were evident throughout the data, such as the recognition of rugby as being highly important to the players but also viewed as ‘just a game’, the expectation of players to be strong yet open to being vulnerable when discussing their wellbeing, and the importance of players’ contribution to the team whilst putting their own needs and performance first before helping others (‘*they would probably put their own life jacket on first… which you are supposed to do*’, RPI Staff 4).

### Rugby: ‘fickle and all-encompassing’

This theme has three subthemes capturing the contextual influences on player mental health and the reality of life as a rugby player, especially the ‘all or nothing’ nature of professional rugby. This theme captures a common discussion throughout the data (i.e., the challenges of elite rugby), highlighting the importance of understanding participants’ social context. More specifically, a need to understand the challenges faced by players in managing their mental health and in engaging with mental health support and interventions (such as the social norms intervention the players engaged with in the present project).

#### The sport of rugby.

This subtheme reflects the lives of elite rugby players in a professional sporting environment, including how on-field performances and external pressures affect the players and their wellbeing. Implicit to the data was a sense of how certain realities of elite rugby and the pressures on players are often accepted as just ‘part of the sport’. This theme highlights the competing demands placed on players, particularly the pressures to remain focused on their on-field performance, which may affect their engagement in intervention workshops such as the ‘5Rs’ which may not be viewed as immediate a priority compared to sporting performance and physical training. The players openly acknowledged the unpredictable nature of life as a professional, their relatively short-term careers, and the potential for rugby to take over other aspects of their lives:

*Your interactions with rugby can be quite fickle and all-encompassing ‘cause it is like a full-time job for players in Ireland and it’s not like a long stable thing (…) there’s like, what 20 or 30% or more of the squad each year would be like actively either having to find like new employment within rugby or change career.* (Player 1)

*I suppose rugby like takes up a large part of our lives in general (…) so it’s hard to then focus down on anything individually.* (Player 3)

Players and staff recognised the working environment in the professional rugby clubs is unique and stressful in its own right (‘*the general mood of the place can be quite all-encompassing*’, Player 4), particularly considering the week-to-week pressure to perform publicly to a high level (‘*it’s a very complicated, I think, place to kind of work and exist and perform week in, week out’,* RPI Staff 1). Part of this challenge for players was juggling their friendships and relationships with teammates who are also their colleagues and competitors for starting positions, even potential competition for future contracts (*‘it’s not just like a straight up normal workplace where you sort of are just colleagues’*, Player 6). In the context of our social norms-based intervention, this raised an early tension in the data in terms of team norms and social functioning in that teammates may have roles as friends and colleagues but also be direct competitors for starting positions on-field and for future contracts.

Both off-field events and on-field performances could have a major impact on the mood and wellbeing of the players and the general atmosphere in the team.

*Everything that could have gone wrong, like rugby-wise, sort of did. So that kind of meant people were quite tense a lot of the time.* (Player 2)

*Results can play a part on like everyone, when everyone’s sort of in either very good or very bad sort of place.* (Player 12)

This was something staff were acutely aware of, with some clubs experiencing particularly challenging years with various off-field and on-field successes and disappointments which had significant effects on team morale and the working social environment for the players:

*I think that it can certainly be affected by major changes in in the environment, whether they’ve won a major trophy or had a huge disappointment or even as we’ve suffered a couple of tragedies within the group.* (RPI Staff 4)

To help manage the pressures of this unique working environment, players discussed the importance of boundaries and especially having a life beyond rugby (‘*it’s something I work on a lot personally, trying to separate what’s going on in here from home’,* Player 3). Whilst rugby is a team sport, it was acknowledged players sometimes need to prioritise their own performance and wellbeing, potentially having clear boundaries between themselves and their teammates to focus on their own performance and wellbeing. This highlights a tension inherent in our intervention, that players may need to prioritise their own wellbeing over helping team-mates given the implications for their own personal wellbeing and performance. This point was viewed as a necessary evil in elite sport:

*Athletes are selfish, you know, they’re going to, they’re gonna put themselves first (…) When someone is having a tough time,* (the) *boys are probably trained to stay away from them because they don’t want to be dragged down with it.* (RPI Staff 4)

The all-encompassing and often unpredictable nature of elite rugby poses various challenges for players to navigate in terms of their own mental health and performance, their relationships with teammates, and in managing the broader sporting environment. Tensions arise between looking after one’s own wellbeing and performance, given competition for starting places and contracts, whilst supporting teammates who are also friends and colleagues, reflecting some complex social relationships within teams.

#### Club culture.

Both players and RPI staff commented on the significant differences in the cultures and working environments across the Irish rugby teams. Broader differences in the cultures of the men’s teams versus the women’s teams in terms of mental health experiences and help-seeking behaviours was observed by the RPI staff especially (noting the women’s teams were not specifically sampled in the project). The men’s players were viewed as being less forthcoming and less open in terms of discussing their mental health with their teammates:

*With the men (…) they may be having the conversations, but they don’t show that they have those conversations. Whereas the women are very, very open.* (RPI Staff 3)

There were also differences in team cultures across the men’s provinces, which was identified by the RPI staff in terms of their approach to developing mental health support (‘*the Ulster environment might be different to Leinster. So, let’s take those nuances into account*’, RPI Staff 5). The coaches and other club support staff also had a major influence on setting the standards, ethos, and environment at their respective clubs. Club staff could, however, be a negative influence on players’ emotional states and wellbeing (‘*coaches could be the cause of some players bad moods*’, Player 11). Players were concerned about disclosing personal mental health concerns to coaches for fear of being dropped, although, one of the club coaches took a different perspective:

*I know from certainly one of the provinces, one of the team coaches would have said that actually that’ll make me more likely to pick you because I know that you’re proactively dealing with something that can get in the way of your performance, whereas if I think you’re anxious and you’re not doing anything about it, then I’d be less likely to pick you.* (RPI Staff 3)

This more open approach facilitated the number of players accessing RPI’s wellbeing service (‘*the highest number of referrals that have come in have been from that province*’, RPI Staff 3). Coaches were recognised for their role in creating supportive cultures and team environments which promote player wellbeing (‘*I think credit to coaches (…) they’ve created a really good environment*’, RPI Staff 4). However, due to the nature of rugby as a professional sport, coach turnover may pose challenges to creating a stable environment which promotes player wellbeing:

*Coaches come and go a lot (…) those guys around the outside, your head coach, your director of rugby are who you need to approach and see (…) can you create something that will always be there, no matter what personnel go through the place.* (RPI Staff 4)

Whilst coaches may be important to setting standards, other key club management and staff may be important to engage in this type of mental health activity particularly in making interventional efforts sustainable and influencing the broader ethos at a club. An understanding of the unique culture and characteristics of each team is needed to successfully design, tailor, and engage players in mental health interventions. In terms of our intervention, not involving the club staff in the workshop discussions appeared to facilitate open player-led conversations about mental health and help-seeking. Although, given the influential nature of club staff and coaches on player wellbeing and the cultures within teams, involving such staff in future mental health interventions may be important for understanding normative influences on player wellbeing and help-seeking and in promoting positive working cultures.

#### Player mental health.

This subtheme describes players’ pre-intervention mental health experiences, the support players seek and provide to one another, and the broader context of player wellbeing in elite rugby. This theme, and the related discussions in the data and in the workshops, highlighted the importance of understanding existing mental health and wellbeing challenges faced by players (as featured in the ‘*Recognise’* part of the 5Rs workshops and posters). Players, when they did access RPI’s mental wellbeing service via referrals from their PDM or the clinical service staff, typically sought help for negative experiences.

*I know from the referral reasons, and from talking to them, it does tend to be mostly around kind of stress, anxiety, and the phrase that they use more than anything else is overthinking. They can’t stop overthinking.* (RPI Staff 3)

Help was typically sought by players in response to a problem rather than focus on bolstering or protecting their mental health (‘*They never look at the positive side*’, RPI Staff 3). Players similarly acknowledged they made use of the RPI’s wellbeing support to manage negative feelings and frustrations.

*I’ve used [name redacted] in here loads like, sometimes I was going out to vent to him for a while and I felt much better afterwards.* (Player 9)

Players were still reluctant to seek help and there was a sense that acknowledging and seeking help for mental health challenges remained a source of shame or embarrassment.

*I think like with all this stuff in general, like with players and men in general, probably takes a lot for you to actually feel like you actually have to reach out.* (Player 3)

Players’ reluctance to seek help may not be specific to their status as professional athletes but may reflect broader perceived norms associated with men and masculinity and the perceived need to be self-sufficient (‘*I definitely think players took it you know on their own, you know, in their own hands’*, Player 2) and the potential shame associated with seeking help (‘*I don’t know if there’s still a stigma around maybe lads not wanting to make a massive deal of it*’, Player 14). This appeared related to the players’ mental health literacy levels around help-seeking, which may not be as developed as initially presumed (‘*Sometimes we assume players know more than maybe we think they do’,* RPI Staff 5). This subtheme had some conceptual similarity to the *‘Refer’* ‘R’ in the workshops and poster, in terms of players’ understanding and experiences of seeking help for mental health concerns and engaging with professional support such as RPI’s wellbeing service.

The ‘*Rugby: ‘fickle and all-encompassing*’ theme highlights the importance of understanding the broader context and influences on player mental wellbeing when developing interventions in elite sport settings. In this case, an understanding of the unique pressures associated with professional rugby, the tensions between individual athlete wellbeing and performance with that of the wider team, and the intense but often very time-limited nature of a professional rugby union career. A further tension lay in players’ need to focus on their own wellbeing and performance, without being affected by other players’ wellbeing challenges, yet still being supportive to teammates who may be friends and colleagues but also competitors for starting positions on-field and for future contracts. Understanding the social psychological functioning of the target population, including the nuances across groups (i.e., the differences across sports teams), is particularly key when developing social norms-focused interventions like our ‘5Rs’ intervention, and in ensuring norms feedback is appropriately targeted and delivered [15].

### Trust and brotherhood

This theme underpinned both the first and third themes and was central to understanding the broader influences on player wellbeing as well as their experiences of the ‘5Rs’ intervention. Staff and players discussed the importance of players’ sense of psychological safety when discussing mental health and wellbeing challenges, when seeking help from teammates and staff, and in the context of developing wellbeing interventions. Throughout the data, players and RPI staff used the word ‘brotherhood’ to describe a sense of shared social identity and group cohesion in the teams beyond merely being colleagues.

*That’s a value a lot of them [the players] would have, that idea of teamship or brotherhood or whatever you want to call it.* (RPI Staff 2)

These bonds and familiarity (*‘we all know each other quite well’,* Player 5) mean players quickly detect when a teammate may be struggling:

*It’s very easy to see maybe when they are doing well … if they’re struggling with something at home or whatever.* (Player 8)

The importance of ‘trust’ that other players would not betray mental health disclosures was viewed by the participants as being key to obtaining support and engaging players in wellbeing workshop activities, including during the present intervention project. This, however, meant many players would seek support from their teammates for ‘smaller stuff’ and may be wary of approaching more formal support:

*I think it’s easier to chat to other lads that may have gone through something similar, rather than facing the reality I suppose in a way, and going to a GP or something* (Player 6)

Players emphasised the importance of not betraying trust when supporting teammates, even if they were concerned about another’s wellbeing and needed to inform a more formal source of support like a GP, their PDM, or RPI’s wellbeing service:

*I don’t really picture myself like doing this unless someone was specifically asking me to* (Player 2)

Maintaining players’ trust when they sought help was also discussed by the PDMs, who are independent of the club coaching staff, in terms of maintaining trust within the team and externally with RPI:

*You’re not letting a coach know about it or whatever because they may not trust you after that.* (RPI Staff 2)

This sense of trust is important given the nature of the competitive rugby sporting environment and the potential short and longer-term consequences of disclosures for players’ opportunities in the sport.

*[players] don’t want to reveal too much about themselves or their vulnerabilities, and where they go for that help could that be detrimental to selection, detrimental to contracts* (RPI Staff 4)

Creating safe spaces for players to express themselves, and potentially be vulnerable, was viewed as key to any mental health focused work in the sport (‘*RPI is independent of their employer so there’s an implicit degree of safety’,* RPI Staff 3). Building and maintaining that sense of ‘trust’ for athletes in team sports, where group bonds (brotherhood or sisterhood) are highly valued, needs factoring into the design of mental health interventions for elite athletes to promote a sense of psychological safety, particularly in the Irish rugby context. This sense of ‘teamship’ (or ‘brotherhood’ in this study) may be an opportunity for group-based interventions to challenge or reinforce existing cultures and promote more psychologically safe, trusting, and supportive environments for players. Whilst this sense of ‘trust’ and ‘brotherhood’ was important throughout the data, this concept mapped specifically to the *‘Reach Out’*, *‘Remain Supportive’*, and *‘Realities of Rugby’* ‘Rs’ in the intervention structure.

### Experiences of the 5Rs of Rugby intervention

The final theme explicitly focuses on the experiences of staff and players in the development and delivery of the 5Rs intervention, the engagement and involvement of players, and a consideration of future developments to support player mental health and help-seeking. The intervention consisted of mental health literacy workshops in each province, led by RPI clinical staff and supported by the province’s PDM. The workshops featured player discussions, activities, and the social norms feedback based on a prior survey of the players’ help seeking behaviours and perceptions of teammates’ help-seeking support (relating to intentions to support a teammate informally and referring a teammate to more formal sources of support). The final part of the workshop was the co-development of a poster resource unique to each province based on the aforementioned ‘5Rs of Rugby’, which were later printed with the logo and player images for that province and displayed in communal areas at the club.

#### Intervention delivery.

The 5Rs project purposefully built on RPI’s existing Tackle Your Feelings campaign (TYF; https://www.tackleyourfeelings.com). TYF is a public campaign featuring professional rugby players as role models and ambassadors, who openly share their mental health stories to help normalize these experiences and had received substantial public attention and had high awareness amongst the provincial clubs’ players. Since 2018, RPI has been incorporating TYF’s messages into their mandatory mental wellbeing education workshops. The RPI staff recognised that the 5Rs intervention ‘*builds on the work that we have done with the Tackle Your Feelings*’ (RPI Staff 1) and ‘*was a good start*’ (RPI Staff 3). Players and staff commented that a collaborative, co-produced, approach used as part of the 5Rs workshop and poster development was novel.

*It’s not really something that would have been a thing until the last year or two.* (Player 1)

*This was just something that we tried that was a little bit new, a little bit different.* (RPI Staff 5)

A specific novelty was the inclusion of social norms feedback in the workshop which was based on a previous survey of the players. The feedback highlighted the players’ very strong intentions to support teammates informally and formally (via signposting to a GP or the RPI wellbeing service) and strong perceived norms of teammates’ behaviours (‘*it’s such an astonishingly high rate*’, RPI Staff 2).

*Like that’s 91% of players would personally refer a player to a formal source of support (…) it being higher than what we would have expected isn’t necessarily a bad thing. It’s just sort of that’s not, that’s not what I would have guessed.* (Player 1)

The feedback indicated no clear difference between what players would personally do or what they thought most of their teammates would do in terms of supporting others’ mental health (‘*The lack of a gap between what they thought versus the reality is interesting’,* RPI Staff 2). As discussed earlier, this lack of a gap between perceived and actual norms was unexpected given the tendency for protective behaviours to be underestimated [15].

*The players believe that their teammates would help them, you know, and the teammates say that they’ll help them.* (RPI Staff 5)

Whilst the novelty of the 5Rs approach was acknowledged by both players and staff, there was consensus that such one-off interventions are not sufficient for facilitating longer-term changes in culture and player wellbeing *(‘If you were to do it again, it would have to be more regular’,* Player 10).

*We’re long enough in this game to know that one intervention is not going to make a difference.* (RPI Staff 5)

*It’s a snapshot in time within each province, so things might be going really well in one province and there might be another province might be having a difficult time.* (RPI Staff 4)

The 5Rs approach was helpful in improving the visibility of RPI’s clinical staff and embedding the wellbeing service players may contact should they require mental health support.

*[Players may say] I remember your man, he was alright. You know that sense of human connection that you don’t get from paperwork or social media.* (RPI Staff 3)

This was particularly important considering COVID-19 imposed limitations on face-to-face interactions in the two years prior to the workshop delivery. Embedding RPI staff in the activities facilitated a sense of trust and psychological safety between the players and staff (building on the second theme), especially as the players have existing relationships with their local PDM who are based at their clubs (*‘I think having the PDMs present definitely helps because there’s a there’s a familiarity and the trust’,* RPI Staff 3)*.*

The province’s 5Rs poster was displayed to the players and RPI staff during the focus groups as a prompt for discussion. Players, however, had mixed opinions on the effectiveness of the posters they co-produced (‘*I probably couldn’t even tell you one of the Rs to be honest with you, but like we’re aware of the stuff there’*, Player 13), although the tailoring of the posters to each provincial club’s identity was better received (‘*I like the photo of the team and stuff*’, Player 1). These posters were displayed by the PDMs in different locations at the provincial clubs, but players had mixed awareness of the posters on-site:

*I know they’re there, but I wouldn’t be noticing them too often*. (Player 11)

*I put the poster up with the players (…) go on where do you wanna put them up around here? (…) in front of the coffee machine, which is the most used machine in the building, was the number one place*. (RPI Staff 1)

Several players, however, described the intervention messaging as getting ‘diluted’, partly as the posters were visible on-site for a while and other mental health activities had been delivered by other organisations.

*I’ve read the 5Rs quite a lot because the poster is just above our coffee machine, so it’s probably on my radar properly (…) it’s been there for so long, probably just diluted like.* (Player 10)

*There’s different groups giving us different talks (…) we’re having too many of them, I think, it’s just getting a small bit diluted to be honest.* (Player 8)

The use of static posters, which displayed all 5Rs at the same time, was a possible issue, with suggestions to cycle the content to better engage players (‘*change [the featured R] every couple of weeks’*, Player 10). Clearly, such print-based materials need careful consideration in terms of managing ‘exposure’ to intervention messaging and avoid messaging losing power, or becoming ‘diluted’ over time. Social norms intervention messaging in particular may require repeated ‘doses’ in order to promote positive behaviours and sustained behaviour change over time [39,40]. The relatively short duration of the current ‘5Rs’ intervention is a likely limitation as raised in the qualitative data presented here.

Displaying the 5Rs posters in team facilities did flag up some resistance and identified some concerning club staff attitudes towards supporting player mental health. This highlights the importance of engaging the broader club staff in these activities given their influence on team functioning, cultures, and norms, and to get their ‘buy in’ to such intervention projects.

*The Academy Manager saw the posters on my desk and was like, can I take two of those (…) when they went to put the poster up, they got push back from one of the people that reports to them, saying: “what are you doing? You know, none of us are qualified to speak to people about their mental health”.* (RPI Staff 2)

Despite these challenges, the intervention package (workshop and 5Rs posters) was generally well received, engaged the players, but there was a clear need to make such work more engaging over the longer term and embed more actionable, prevention-focused activities to support player wellbeing. One of the PDMs summed up the staff focus group discussions, the team environments, and the wider benefits of professional rugby:

*There’s a lovely bit of evidence that when you intervene early, they’re prepared and proactive about dealing with issues. I just think it would be a really big selling point to why, if you’re picking between different sports to participate in, this is a great environment to be in.* (RPI Staff 2)

#### Engaging and involving players.

Key to player engagement and involvement in the 5Rs workshop activities was its focus on discussion and co-production of the posters with players, which aimed to promote engagement with the intervention content.

*I suppose we’re constantly trying to come up with something new every year rather than just roll out the same old presentation on mental health literacy to players. So, we try and make it different for them and get them involved.* (RPI Staff 1)

Some players were reluctant to participate (‘*initial reaction you can get from players is not again, we already know this*’, RPI Staff 1). Others were uncomfortable with the workshop’s focus on mental health literacy (specifically featured as part of the ‘*Recognise’* ‘R’), including the presentation of statistics on the prevalence of common mental health conditions and the discussions of mental health experiences and support. This was visible through some players not engaging or taking the workshops seriously at first:

*We found them [at one province] quite respectful of each other. Whereas in a different province it was trickier to manage the giggling and messing.* (RPI Staff 3)

This noticeably changed as the workshop focused on more serious mental health matters.

*When we started talking confidentiality and mentioned the word suicide [it] immediately got their attention. Oh, hang on, we’re talking about serious shit here.* (RPI Staff 3)

Such experiences were not common across all the provinces, with the observing staff noting the creation of a safe environment for the players to discuss mental health-related experiences and issues. This reinforces discussions in the previous themes relating to the importance of ‘trust’ and ‘psychological safety’, and in understanding the different social environments associated with each club (including the team culture, norms, and working environment).

*They* (the players) *took it very seriously to be fair to them. And I was blown away by the environment, first of all, that was created for them, and I suppose safety, for lack of a better word, to be able to say those things.* (RPI Staff 4)

There were several factors which improved engagement in the workshops, particularly the influence of respected role models in the teams and the engagement of more senior players in the discussions which permitted the more hesitant younger players to participate (‘*the older players were more psychologically literate than some of the others*’, RPI Staff 3). This was somewhat of a surprise considering the assumptions that younger players would be more literate in terms of their mental health understanding given broader societal changes of increasing mental health awareness in younger age groups. Although, previous research with elite rugby league players has reported a similar relationship between improved mental health and older age [6].

A separate challenge with delivering this intervention was accommodating the workshops in the players’ schedules, with workshops scheduled later in the day after players had completed physically demanding training sessions (*‘You usually get them at a time at the end of the day and they’re tired’,* RPI Staff 5), with club coaches identified as a barrier.

*If you did this at the start of a day you’d get more engagement, but I don’t know if the rugby coaches would allow you.* (Player 6)

*The feedback I’m actually getting now is can you please stop putting stuff at 3pm on a Tuesday cause you’re taking away from the day’s learning of on-field stuff by tagging something in (…) they’re already really tired now, you’re mixing messaging. It’s valuable, but it’s not part of the programme.* (RPI Staff 2)

Alongside greater player involvement and co-production, there clearly remains scope for greater involvement of club staff (coaches and other off-field staff) in developing and producing such interventions, which would help further promote player engagement.

#### What’s next?.

Several suggestions for how the 5Rs intervention could be developed were offered by players and staff. These suggestions particularly focused on embedding activities focusing on prevention and upskilling players to support teammates in practice (‘*how to action some of this*’, RPI Staff 4), indicating further opportunities to develop the *‘Reach Out’* and *‘Remain Supportive’* components of the intervention.

*I think a lot of it everywhere is about what to do if something bad happens instead of how to manage yourself.* (Player 7)

*The more opportunities we can give players to learn the language around having these difficult conversations [the better].* (RPI Staff 1)

This is important given the players were generally sceptical towards mental health workshops, viewing these as being reactive in nature rather than being proactive in terms of supporting their mental health (‘*it seems to be all quite reactive (…) even as players we are probably a bit reactive*’, Player 8). Players also recognised a need to further promote a supportive culture in the clubs and promote help-seeking for a range of experiences (‘*it’ll help a lot of people, obviously outside rugby too, which is which is important’*, Player 7). This is particularly important in the *‘Realities of Rugby’* ‘R’ in terms of further normalising such experiences and help-seeking behaviours within teams.

*If there’s any sort of avenue of creating a culture where it’s like it doesn’t necessarily have to be like someone’s really struggling to go and see someone* (Player 3)

A key benefit from the 5Rs project was improving staff understanding of players’ mental health literacy (specifically in ‘*Recognise’* but also throughout the intervention). This understanding could then be integrated into other support for players (‘*Some of the language from 5Rs could be integrated into that daily check in’*, RPI Staff 1) and be tailored to the players and the wider clubs.

*Some of what they came back with about how they want to be approached has informed how I have conversations with them now* (RPI Staff 2)

*What we have from each of the provinces is their language around what mental health and wellbeing looks like in their environment and how they want to react.* (RPI Staff 1)

In summary, players and staff identified a number of strengths and areas to develop based on their experience of participating in, and co-producing, the 5Rs intervention (workshops and posters). We have provided a discussion of the themes identified through our reflective thematic analysis in relation to each of the ‘Rs’ which formed the basis of the intervention approach. The ‘5Rs’ themselves were generally positively received as concepts and provided a clear structure to the intervention; however, there are opportunities to further develop each of these ‘Rs’ in practice (e.g., through providing more in-depth activities and guidance framed around each ‘R’ in turn). As indicated by our analysis, there is a clear need to understand the social psychological functioning of elite sports teams when developing such intervention approaches. The present study’s focus on elite rugby union did highlight a number of contextual, (social) environmental and social psychological factors to consider when developing work of this nature, specifically the group dynamics within teams, team cultures and social norms which may substantially differ across teams within the same sport.

## Discussion

This study provides a novel qualitative exploration of staff and players’ experiences of a social norms and mental health literacy intervention focusing on social support and help-seeking in the elite rugby provincial teams in Ireland (‘*The 5Rs of Rugby’*). This is the first project to develop and conduct a Social Norms Approach (SNA) intervention in an elite performance setting, co-producing elements of the intervention with professional athletes, and is one of few SNA interventions focusing on mental health literacy.

The findings of this qualitative exploration highlight the importance of understanding the contextual nature of elite sport when developing mental health interventions for athletes (‘*Rugby: fickle and all-encompassing’* theme) [41]. Careful consideration of the wellbeing challenges associated with high performance settings, the varied off- and on-field pressures on player wellbeing (e.g., competition for contracts and starting positions), and the unique group dynamics, social norms, and cultures in elite sports teams is required when developing such mental health-focused interventions. We integrated these contextual influences as the fifth ‘*R*’ in our ‘5Rs’ intervention, extending a previously developed intervention framework used with staff working in an elite sports network [17]. This fifth ‘*R*’ (‘*The Reality of Rugby’*) featured explicit social norms feedback on the perceived versus actual intentions to support teammates’ mental health, based on the established Social Norms Approach [15,42] and our prior work in other settings [28], and building on Sebbens et al.’s intervention workshops which only briefly focused on staff perceptions of player help-seeking behaviours [17]. The lack of a norms gap between players’ personal behaviours and perceived teammates support via formal and informal routes was noteworthy, with no evidence of the underestimation of the norms found for other health-protective behaviours [15]. Instead, players reported strong intentions to support teammates and demonstrated no clear underestimation or misperception of team norms, reflecting the strong interpersonal relationships amongst the players and the supportive cultures at the provincial clubs.

A sense of ‘*Trust and Brotherhood*’ and the psychological safety this gave players was an important influence on wellbeing, team functioning, and the intervention delivery [41]. Creating a safe space for discussing and disclosing mental health experiences was identified by players and staff as a strength of the 5Rs workshops, helping to normalise mental health conversations and increasing the visibility of the RPI staff. RPI’s existing Tackle Your Feelings campaign has assisted in reducing mental health stigma, which when combined with the positive social environment created during the 5Rs intervention (including reinforcing positive social norms, promoting team cohesion, and reducing the fear associated with potential disclosures), facilitated a sense of psychological safety amongst the players [43]. Psychological safety has been identified as a key factor in enhancing player health, team functioning and performance [44].

In terms of the development and evaluation of the intervention (‘*Experiences of the 5Rs of Rugby intervention’)*, the workshops and 5Rs posters provided an important insight into players’ mental health literacy and the language they use to describe their personal mental health experiences, something which the RPI staff have since integrated into their own practice. There was, however, consensus across the data that the 5Rs language requires greater embedding into the players’ day-to-day activities (e.g., raising player awareness by changing ‘*R*’ focus over time), that ongoing support beyond one-off interventions is needed, with a greater focus on prevention and skills-based practical activities so players know how to seek help and promote their own, and teammates’, mental wellbeing. Providing players with activities that they can rehearse, like their on-field drills, was one suggestion for increasing confidence when seeking support and supporting others.

One potential avenue for future Social Norms Approach interventions in elite sport settings is to increase the involvement of players in the intervention design and delivery, akin to a more explicitly ‘co-produced’ approach. We were limited in terms of time and resources to take a fully ‘co-produced’ approach in the present project, but greater involvement of players and club staff in the intervention development and delivery may increase engagement with the project. SNA interventions in other applied settings have made effective use of steering and advisory groups to engage the target population and guide intervention development [28,31], alongside a thorough understanding of the target group’s social environment and likely effective intervention components [45,46]. Greater player involvement may address some of the issues our qualitative evaluation has highlighted, especially players’ scepticism of mental health interventions and reluctance from some players to engage with the intervention content.

Integrating the 5Rs messaging with RPI’s existing Tackle Your Feelings mental health literacy campaign could also be considered to further promote player awareness and reinforce messaging over time. Greater buy-in and support from coaches and club staff may also be needed to further promote player engagement, such as providing appropriate time for mental wellbeing workshops in players’ schedules and placing greater importance on player mental wellbeing alongside their physical wellbeing and performance. Despite the mental wellbeing of players being a key influence on on-field performance, some coaches may still view such work as being ‘*valuable, but it’s not part of the programme’*, as highlighted by one of the RPI staff. Greater engagement from key club staff is required for such interventions to be effective in the future.

In terms of the broader literature, surprisingly few interventions focusing on mental health literacy and help-seeking in the context of professional rugby have been published. Mental health-related intervention studies have tended to focus on specific groups of players, often youth or academy players [47,48], not necessarily full professional players in the public eye. There are a number of intervention studies that focus other aspects of elite rugby, particularly concussion and other head injuries [49,50], and so there remains a gap in terms of intervention work supporting player mental wellbeing. We are unaware of similar social psychology-grounded interventions similar to the work we report here with professional rugby players. The 5Rs project provides a novel interventional approach for promoting mental health and help-seeking amongst current active professional rugby players, although further intervention work and development of our approach is warranted as discussed in our analysis.

There are some strengths and limitations to consider with the current qualitative evaluation of players and staff experiences of participating in the intervention project. First, we were only able to conduct focus groups with men’s players from two of the provinces and it may be that players from other teams have different views and experiences of the delivered intervention and of player wellbeing more broadly. For various reasons we were unable to include the Irish Women’s 15s and 7s teams in the wider intervention project (see [16] for further details). Whilst the rich qualitative data presented here has identified some key differences across teams in terms of their mental health experiences and behaviours, and in staff experiences of working with the men’s and women’s teams, the intervention approach may have been experienced differently across rugby codes and groups. Although, we did collect rich data from players and RPI staff regarding the development and delivery of the intervention which yielded robust themes grounded in the data, which map onto the different 5Rs as highlighted in the analysis. The novel findings presented here represents one of the few qualitative studies of participants’ experience of engaging with SNA interventions and is the first such intervention to be conducted with elite athletes in a competitive sporting environment.

## Conclusions

Our qualitative evaluation of the player and staff experiences of developing and participating in the 5Rs of Rugby project identified several key considerations for conducting social norms and mental health-focused interventions in elite sport settings. First, there needs to be an understanding of the context and lived experience of the target group [41]. In this project, an understanding of the influences on professional rugby players’ mental health and team functioning, particularly players’ need for psychologically safe environments that support mental health disclosures without fear of harmful consequences, was key [43,44]. Second, there needs to be a careful scaffolding of intervention messages to improve mental health literacy and to promote positive social norms (in this case, reinforcing players’ existing highly positive mental health support and help-seeking norms) [15]. Finally, consideration of how to build longer-term help-seeking and mental health support is required whilst factoring in the unique group dynamics and cultures in elite sports teams. Ensuring the buy-in from coaches and other club staff is important for promoting healthy team environments and the sharing and disclosure of mental health concerns amongst athletes. The interventional approach evaluated here represents an advancement of an existing mental health literacy framework [17]; however, developing mental health literacy education for staff and players using frameworks like the ‘5Rs’, and considering the contextual influences on athletes, should be part of the development work in future intervention efforts given the success of this approach in this project.

## Supporting information

S1 Text Topic schedules for qualitative evaluation. (DOCX)
