# Supplementary material for: The 5Rs of Rugby: A qualitative evaluation of the development, delivery, and experience of a mental health literacy and social norms intervention with elite rugby union players in Ireland
Source: PLOS Ment Health. 2025 Sep 24;2(9):e0000258. doi: 10.1371/journal.pmen.0000258 (PMC12798509; doi:10.1371/journal.pmen.0000258)
Supplement: S1 Text — (DOCX) [file pmen.0000258.s001.docx]

**Supplementary File 1 – Topic Schedules for Qualitative Evaluation**

**Topic Schedule – Focus Group Discussions (Players)**

Researcher welcomes the players to the discussion and briefly outlines the focus group ground rules (i.e. information shared is not to be shared outside of the group, a recording of the discussions will be made and anonymised later, anonymous quotes may be taken from the transcripts which will not identify any specific player, players should say if they want any comments they’ve said removed from the transcript).

**RECORDING STARTS**

Researcher: Thank you for agreeing to take part in this focus group discussion, before we start the discussion, please could you confirm your consent by speaking on the microphone or typing ‘yes’ in the text box.

Researcher: The aim of this focus group discussion is to get your experiences of taking part in the Tackle Your Feelings workshops and the overall project on player mental health and supporting team-mates. These focus groups are your opportunity to discuss your experiences and any feedback you have about this project (positive and negative). To recap, you will have had the opportunity as a player to take part in three mental wellbeing questionnaires that focused on your wellbeing throughout a season and to take part in a workshop that outlined the 5Rs of Mental Wellbeing in Rugby.

1. What were your overall experiences of taking part in this project?
2. As part of the project, each workshop developed their own ‘5Rs of Rugby’ poster – what did you think of these 5Rs? (Researcher presents each group’s 5R poster on screen)
   1. What did you think of the messages on the poster?
   2. Has developing this poster changed how you view yourself/team-mates?
3. What did you think of the social norms message featured in the workshops? (Researcher displays the social norms feedback presented in the workshops)
   1. What did you think when you saw these norms messages in the workshop?
4. Have you noticed any changes in your team after these workshops? (e.g. amongst your team-mates)
   1. Any changes in team morale / supporting each other? (How/Why?)
5. If we were to do this type of project/intervention again, what could be improved?
   1. What should we keep doing if we did this workshop/intervention again? Anything didn’t work?
6. Finally, what sort of ongoing support for mental health and wellbeing do rugby players still need? (e.g. additional support?)
   1. Does player mental wellbeing change over the season? (If so, how?)
   2. What sort of education/activities need to be done over the season? (Different activities at different times – what sort?)
   3. How can we encourage more players to use RPI’s wellbeing service?
   4. What support could be done in the dressing room / at your club?
7. Is there anything else you’d like to discuss / share about your experiences of this project?

Researcher thanks the participants for their involvement in the discussion, reminds participants of next steps and withdrawal options (i.e. contact RD within two weeks if wishing to withdraw their data) and stops the recording.

**RECORDING STOPS.** Study ends.

**Topic Schedule – Focus Group Discussions (RPI Staff)**

Researcher welcomes the participants to the discussion and briefly outlines the focus group ground rules (i.e. information shared is not to be shared outside of the group, a recording/transcription of the discussions will be made and anonymised later, anonymous quotes may be taken from the transcripts which will not identify any specific individual, participants should say if they want any comments they’ve said removed from the transcript).

**RECORDING STARTS**

Researcher: Thank you for agreeing to take part in this focus group discussion, before we start the discussion, please could you confirm your consent by speaking on the microphone or typing ‘yes’ in the text box. The aim of this focus group discussion is to get your experiences of facilitating the Tackle Your Feelings social norms feedback workshops and the overall project on player mental health and supporting team-mates. These focus groups are your opportunity to discuss your experiences as staff involved in the project, your impressions of the players’ experiences, and any feedback you have about this project (positive and negative). To recap, players had the opportunity to take part in three mental wellbeing questionnaires focusing and took part in a workshop that outlined the 5Rs of Mental Wellbeing in Rugby.

1. What were your overall experiences and impressions of this project?
2. As part of the project, each workshop developed their own ‘5Rs of Rugby’ poster – what did you think of these 5Rs? (Researcher presents each group’s 5R poster on screen)
   1. What did you think of the messages on the poster?
   2. What were the reactions of the players to these 5R posters?
3. What did you think of the social norms message featured in the workshops? (Researcher displays the social norms feedback presented in the workshops)
   1. What did you think when you saw these norms messages in the workshop?
   2. What were the players’ reactions to the normative feedback?
4. Have you noticed any changes amongst the players/teams after these workshops?
   1. Any changes in team morale / supporting each other? (How/Why?)
5. If we were to do this type of project/intervention again, what could be improved?
   1. What should we keep doing if we did this workshop/intervention again? Anything didn’t work?
6. Finally, what sort of ongoing support for mental health and wellbeing do you think rugby players still need? (e.g. additional support?)
   1. Does player mental wellbeing change over the season? (If so, how?)
   2. What sort of education/activities need to be done over the season? (Different activities at different times – what sort?)
   3. How can we encourage more players to use RPI’s wellbeing service?
   4. What support could be done in the dressing room / at your club?
7. Is there anything else you’d like to discuss / share about your experiences of this project?

Researcher thanks the participants for their involvement in the discussion, reminds next steps and withdrawal options (i.e. contact RD within two weeks if wishing to withdraw their data) and stops the recording.

**RECORDING STOPS.**

Study ends.

**Topic Schedule – Player Online Survey**

Demographics items:

Please state your age in years: ____________

Please indicate which team you belonged to last season: (Drop-down menu of the provincial teams)

Thank you for taking part in this project. The questions below are about your experiences of taking part in the Tackle Your Feelings Workshops and surveys over the 22/23 season, including the workshops where you discussed player mental health, supporting team-mates, created the ‘5Rs of Rugby’ posters, and viewed the feedback about the social norms of supporting team-mates with their mental health and wellbeing.

The below questions are open-ended, so please do share your thoughts and experiences and aim to write a couple of sentences per question.

1. What were your overall experiences of taking part in this project?
2. As part of the project, each workshop developed their own ‘5Rs of Rugby’ poster (An example image of the 5Rs poster will be shown on screen)
   1. What did you think of the posters and the 5Rs messages?
   2. Has developing this poster changed how you view yourself/team-mates?
3. What did you think of the social norms messages featured in the workshops?

(An image of the slide with the norms will be shown on the webpage)

1. Have you noticed any changes in your team after these workshops? (e.g. in player behaviour, mental health, supporting each other?)
2. If we were to do this type of project/intervention again, what could be improved?
3. What should we keep doing if we did this workshop/intervention again?
4. Does player mental health/wellbeing change much over the season? (How/Why?)
5. What sort of ongoing support for mental health and wellbeing do you think rugby players still need?
6. How can we encourage more players to use RPI’s wellbeing service?
7. Is there anything else you’d like to share about your experiences of this project?
